# Outcome of hypofractionated breast irradiation and intraoperative electron boost in early breast cancer: A randomized non‐inferiority clinical trial

**DOI:** 10.1002/cnr2.1376

**Published:** 2021-04-01

**Authors:** Pedram Fadavi, Nahid Nafissi, Seied Rabi Mahdavi, Bahareh Jafarnejadi, Seyed Alireza Javadinia

**Affiliations:** ^1^ Department of Radiation Oncology Iran University of Medical Sciences Tehran Iran; ^2^ Department of General Surgery Iran University of Medical Sciences Tehran Iran; ^3^ Department of Medical Physics Iran University of Medical Sciences Tehran Iran; ^4^ Cellular and Molecular Research Center Sabzevar University of Medical Sciences Sabzevar Iran

**Keywords:** boost, cosmetic outcome, electrons, hypofractionation, intraoperative radiotherapy, toxicity

## Abstract

**Background:**

Intraoperative electron radiotherapy (IOERT) followed by hypofractionated whole breast irradiation (HWBI) provides the shortest possible time of adjuvant breast irradiation. The efficacy of either method has been described in previous reports; however, to our knowledge, the efficacy of combined therapy has not been reported.

**Aim:**

To compare the toxicity and cosmetic outcome of IOERT as a tumor bed boost followed by HWBI with conventional whole breast irradiation (CWBI) followed by external electron tumor bed boost (EETBB) after breast conserving surgery (BCS) in patients with invasive breast cancer.

**Methods:**

In 2019, a prospective noninferiority trial (IRCT20180919041070N2) was started. After BCS, early‐stage breast cancer patients were treated by IOERT (10 Gy) and HWBI (42.56 Gy in 16 fractions) or CWBI (50 Gy in 25 fraction) and EETBB (10 Gy in 5) in a double‐arm design. Acute/late toxicity and cosmetic outcome were evaluated by common toxicity criteria (CTC) after 1‐year follow‐up (FUP) at the level of *p* < .05.

**Results:**

Of 60 eligible patients, 30 were allocated to each group. Regarding acute effects after a median FUP of 12 months, CTC‐score of grade II‐III erythema (*p* = .001) and desquamation (*p* = .005) were significantly higher in CWBI+EETBB compared to IOERT+ HWBI. However, there were no significant differences at the end of radiotherapy and after 1 month, 6 months, and 1 year. Cosmetic outcome after radiation was similar in both groups mostly rating as good/excellent after 1‐year FUP.

**Conclusions:**

Boost‐IOERT/HWBI regimen has comparable acute and late treatment toxicity profiles compared to the CWBI.

## 1 INTRODUCTION

Adjuvant whole breast irradiation followed by tumor bed boost with a total dose of 60 Gray (Gy) is the current standard of care in breast cancer radiation therapy after breast conserving surgery and is usually done by external beam radiotherapy during 6 weeks. Because of the low α/β ratio of breast cancer cells,^1^ hypofractionated radiation therapy regimens utilizing higher dose per fractions up to 2.6–3.2 Gy leading to considerable shorter duration of treatment time is considerably gaining great interest in the recent years.^2^, ^3^, ^4^, ^5^

Two common methods that utilize large dose per fraction in breast cancer radiation therapy include hypofractionated whole breast irradiation (HWBI) and intraoperative electron radiotherapy (IOERT). Both of these methods have been assessed separately in recent studies.^5^, ^6^ However, the role of tumor bed boost in HWBI regimens has not yet been fully addressed but available data support its integration into these regimens in order to decrease the long term in‐breast recurrence rates.^5^, ^7^ Nevertheless the role of tumor bed irradiation boost dose on overall survival is unclear.^7^

Moreover, the outcome and toxicity profile of IOERT followed by HWBI has not been explicitly addressed, and current data come from phase II and/or single arm clinical trials.^8^, ^9^ The first study by Ivaldi et al showed that IOERT of 12 Gy is feasible and has an acceptable acute and late toxicity.^8^ Subsequently, Fastner et al confirmed the safety and tolerability of Boost‐IOERT/HWBI regimen in a larger scale with longer follow up period.^9^ However, a comparative study assessing the noninferiority of Boost‐IOERT/HWBI regimen relative to conventional whole breast irradiation (CWBI) followed by external electron tumor bed boost (EETBB) has not been reported. This study has been conducted to compare the toxicity and cosmetic outcome of IOERT as a tumor bed boost followed by HWBI with conventional whole breast irradiation (CWBI) followed by external electron tumor bed boost (EETBB) after breast conserving surgery (BCS) in patients with invasive breast cancer.

## 2 MATERIALS AND METHODS

### 2.1 Study patients

This was a prospective clinical trial phase II study approved by the Research Ethics Committee of Medical School of Iran University of Medical Sciences (IR.IUMS.FMD.REC.1398.319). The study has been registered on Iranian Registry of Clinical Trials (IRCT20180919041070N2). Written informed consent was obtained from all patients prior to the enrollment. Female patients aged ≥35 years with biopsy‐confirmed invasive breast carcinoma, regardless of the molecular subtypes of the tumor, on pathology were eligible to enroll in this study. Inclusion criteria consisted of tumor size ≤5 cm (pT1‐2), no regional nodal involvement or involvement of ≤3 nodes (pN0‐1), and clear surgical margin in postsurgical pathological examination. There was no limitation regarding the hormone/systemic therapy.

Patients with a Karnofsky Performance Score of ˂70%, previous history of chest wall irradiation, multicentric breast cancer, history of connective tissue diseases including lupus and scleroderma, and large breasts based on planning target volume in radiotherapy more than 2500 ml were excluded. Patients who fulfilled these criteria were included in the present study.

### 2.2 Trial design and definition of primary endpoints

The study was designed as a double‐armed prospective trial. The primary endpoints were defined as treatment tolerance and cosmetic outcome. NCIC common toxicity criteria (CTC) Version. Twenty and the late effects in normal tissues – subjective, objective, management, and analytic (LENT SOMA) scales were used to assess the acute and late toxicities.^10^, ^11^, ^12^ The acute reactions were assessed during the radiotherapy, at the end of the radiation therapy, and in week four post HWBI/CWBI‐EETBB. For late reactions, the assessments were performed after 6 months and a year after the treatment.

Late reactions were classified into two groups; group one that consisted of grade zero and one, group two that included grade two and three. Grade zero and one were categorized in one class and grade two and three in another one. Cosmesis was evaluated by physicians at year one using a 5‐point‐Scoring System which was previously introduced by van Limbergen et al.^13^

### 2.3 Treatment schedule

In the Boost‐IOERT/HWBI group, patients received IOERT of 10 Gy at the time of breast conserving surgery. Postoperatively, HWBI in 16 sessions with a total dose of 42.56 Gy was prescribed for all patients in the supine position using two high tangential fields with 6 MV photons after the completion of adjuvant chemotherapy if indicated. In the CWBI‐EETBB group, after BCS and adjuvant chemotherapy if indicated, the whole breast irradiation was done with similar beam arrangement with dose of 50 Gy in 25 fractions followed by the external electron boost dose of 10 Gy in five fractions. Both HWBI and CWBI‐EETBB were performed by 3D conformal radiotherapy technique.

Clinical target volumes were delineated in both groups based on the breast cancer contouring atlas that has been introduced by Radiation Therapy Oncology Group (RTOG).^14^ The main dose constrains, which were adopted, were V20 of ipsilateral lung and V25 of heart for left sided breast cancers which were kept below 20 and 10%, respectively.

### 2.4 Data registration, quality assurance, and follow‐up

Patients were visited weekly during the radiation, at the end of radiation therapy, and in week four, month six, and year one post HWBI/CWBI‐EETBB.

### 2.5 Statistical methods

Sample size was calculated 30 patients in each group to achieve a power of 90% to detect skin toxicity differences between two groups using *n* = (*Z*_*α*/2_ + *Z*_*β*_)^2^ × (*p*_1_(1‐*p*_1_) + *p*_2_(1‐*p*_2_))/(*p*1‐*p*_2_)^2^ formula considering prevalence of skin toxicity of 66.6 and 24.1% in HWBI with or without boost, respectively.^15^ Data were analyzed by SPSS 11 using Chi‐square, Fisher's exact, and *T* tests at the level of *p* < .05. The data had a normal distribution based on Shapiro–Wilk test.

## 3 RESULTS

**FIGURE 1:**
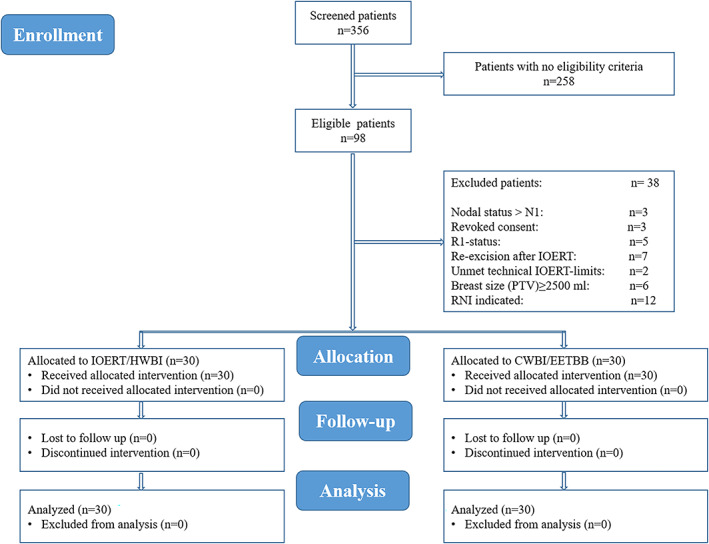
CONSORT subject flow diagram shows the number of subjects screened, enrolled, randomized, and included in the primary analysis

Out of 356 screened patients with breast cancer, 30 patients were enrolled in each group. Figure 1 shows CONSORT subject flow diagram.

**FIGURE 2:**
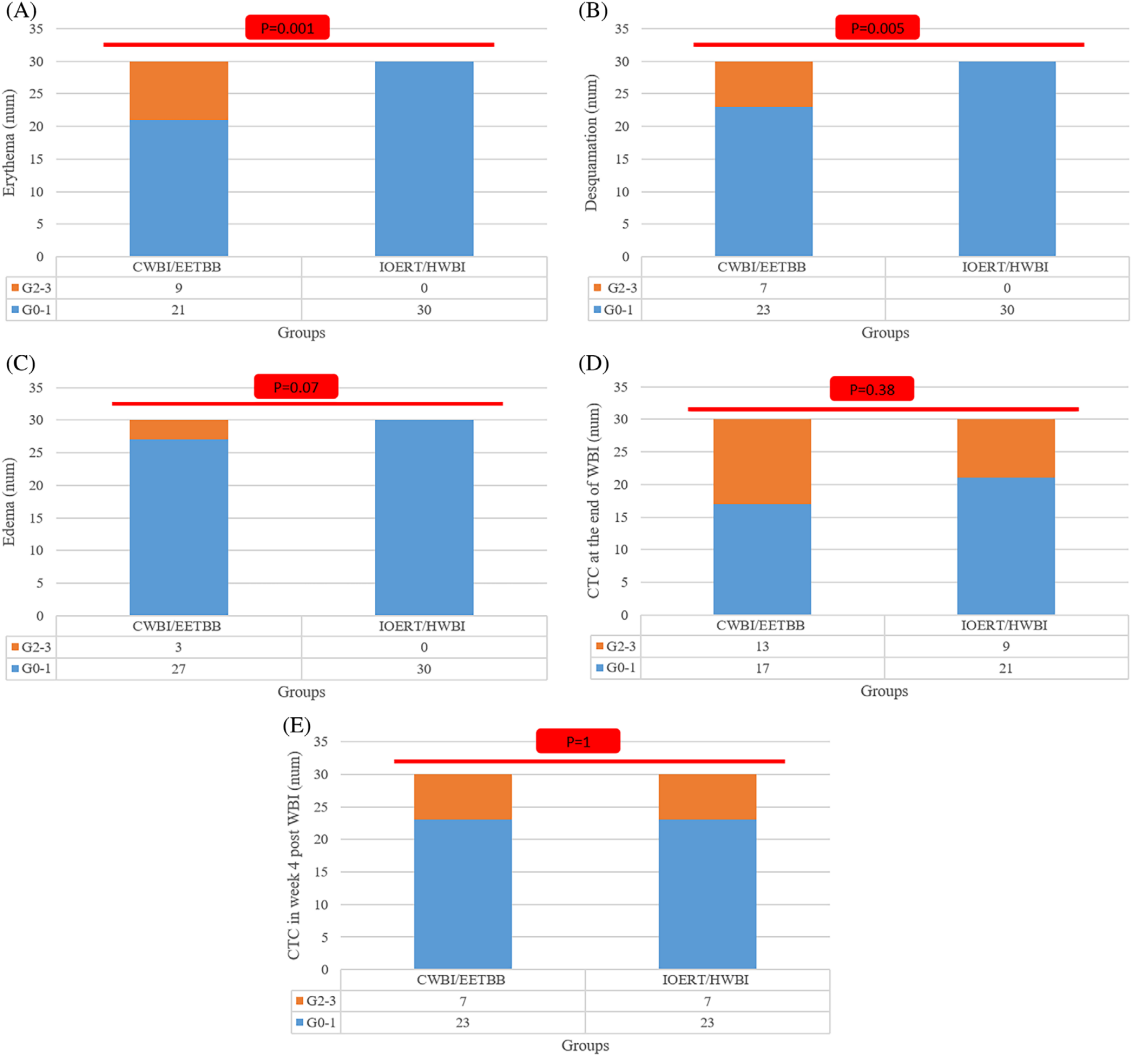
Acute toxicities assessed by common toxicity criteria during WBI (A–C), at the end of WBI (D) and in week 4 (E)

Mean age of patients in IOERT/HWBI and CWBI/EETBB were 47.3 ± 10.8 and 46.5 ± 8.5 years, respectively (*p* = .77). Both groups were similar in terms of laterality, grade, pathologic stage, ER/PR/Her2neu statues, and Ki67 expression. Patient characteristics are shown in Table 1. During the WBI, grade 2–3 erythema and desquamation were significantly higher in CWBI/EETBB group compared to IOERT/HWBI group (*p* = .001, and *p* = .005, respectively). Edema during the WBI (*p* = .07), CTC at the end of the WBI (*p* = .28), and in week 4 post HWBI/CWBI‐EETBB (*p* = 1) were similar in both groups (Figure 2).

**TABLE 1:** Patient characteristics

Characteristics	All *N* (%)	IOERT/HWBI *N* (%)	CWBI/EETBB *N* (%)	*p* Value
Involvement side				
Left	37 (61.7)	19 (63.3)	18 (60)	.79
Right	23 (38.3)	11 (36.7)	12 (40)	
Grade				
I	15 (25)	10 (33.3)	5 (16.7)	.28
II	17 (48.3)	12 (40)	17 (56.7)	
III	16 (26.7)	8 (26.7)	8 (26.7)	
pT stage				
T1	25 (41.7)	18 (60)	7 (23.3)	.0004
T2	35 (58.3)	12 (40)	23 (76.7)	
pN stage				
N0	49 (81.7)	20 (66.7)	29 (96.7)	.003
N1	11 (18.3)	10 (33.3)	1 (3.3)	
pStage				
I	19 (31.7)	12 (40)	7 (23.3)	.16
II	41 (6.3)	18 (60)	23 (76.7)	
ER status				
−	3 (5)	0 (0)	3 (10)	.07
+	57 (95)	30 (100)	27 (90)	
PR status				
−	13 (21.7)	8 (26.7)	5 (16.7)	.34
+	47 (78.3)	22 (73.3)	25 (83.3)	
Her2 status				
−	48 (80)	22 (73.3)	26 (86.7)	.19
+	12 (20)	8 (267)	4 (13.3)	
Ki67				
14%>	15 (25)	8 (26.7)	7 (23.3)	.76
14%<	45 (75)	22 (73.3)	23 (76.7)	

**FIGURE 3:**
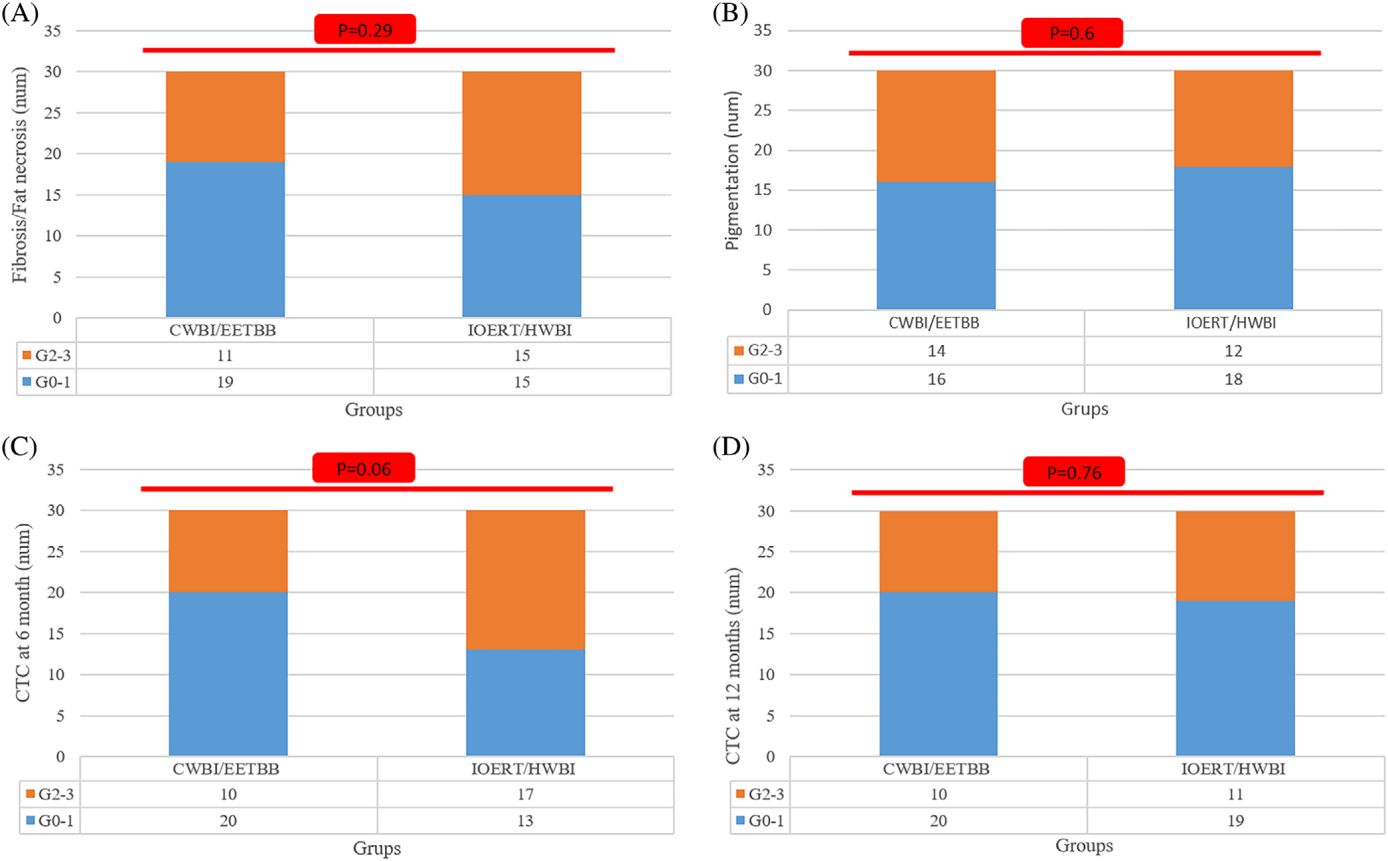
Late toxicities assessed by LENT SOMA scales (A and B) and common toxicity criteria at 6 (C) and 12 (D) months after WBI

In long term follow up, CTC at 6 and 12 months, fibrosis/fat necrosis, and pigmentation were the same in both groups (Figure 3). No grade 4 late toxicity was reported in both groups.

Most of cosmetic objective scores were reported as excellent/good without any significant differences (*p* = .1). No poor/fair cosmesis outcomes were reported in the present study (Table 2).

**TABLE 2:** Cosmetic objective scores in IOERT/HWBI and CWBI/EETBB groups

Characteristics	All *N* (%)	IOERT/HWBI *N* (%)	CWBI/EETBB *N* (%)	*p* Value
Involvement side				
Excellent	20 (33.3)	7 (23.3)	13 (43.3)	.1
Good	31 (51.7)	16 (53.3)	15 (50)	
Moderate	9 (15)	7 (23.3)	2 (6.7	
Fair	0 (0)	0 (0)	0 (0)	
Poor	0 (0)	0 (0)	0 (0)	

## 4 DISCUSSION

This study aimed to compare the toxicity and cosmetic outcome of two different radiation schedules: IOERT as a tumor bed boost followed by HWBI and CWBI followed by EETBB after BCS of patients with invasive breast cancer. The results revealed that acute skin toxicities during the WBI were significantly higher in the conventional group. There was no significant difference regarding the grade II–III toxicities in IOERT/HWBI group at the end of WBI. Late toxicities were similar in both groups with no reported grade 4 toxicity. Cosmesis outcomes were mostly reported as excellent/good in both groups.

Various hypofractionated radiotherapy schedules with the aim of decreasing total dose to 40–44 Gy and shorter treatment duration of 15–16 fractions have been used in recent years. In 2002, Whelan et al reported the non‐inferiority of HWBI in a large‐scale clinical trial for the first time and described the long‐term safety of IORT in patients with breast cancer in whom regional nodal irradiation was not indicated.^2^, ^3^, ^5^ Subsequently, Zygogianni et al assessed the cosmetic outcome of external electron boost to tumor bed in patients with early breast cancer retrospectively. They showed that acute skin toxicity was significantly higher in patients who received tumor bed boost externally in comparison to IORT. The late skin toxicity, however, was similar between the groups.^15^

The safety and feasibility of applying intraoperative electron tumor bed boost followed by HWBI were first described by Ivaldi et al.^8^ They reported that the skin toxicity might be observed in all patients; however, it was mostly grade 1 that peaked at the end of WBI. In long term follow up, less than 2% patients experienced grade 3 or higher toxicity.^8^ In a recent study of 627 patient with early breast cancer, Fastner et al described the safety of applying of IOERT of 11.1 Gy as tumor bed boost followed by HWBI of 40.5 using 2.7 Gy per fraction.^9^ They showed that most of the patients experienced grade 0/1 skin toxicities at the end of the WBI and in week 4 after completion of treatment. In long term follow up, late skin toxicity was reported in up to 3.5% of patients and cosmesis was mostly scored as excellent/good. Our study, as the third trial testing IOERT followed by HWBI, provided further reassurance regarding the safety of this procedure and confirmed its cosmetic outcome.

Biologic and in vivo effectiveness of the intraoperative tumor bed radiotherapy using electron or photon and hypofractionated external radiation regimens have been previously proven separately in different scenarios such as partial breast irradiation and/or as a part of adjuvant treatment.^16^, ^17^, ^18^, ^19^, ^20^ However, the role of tumor bed boost, regardless of its method of application (like intraoperative, brachytherapy, and external technique) has not been fully described,^5^, ^7^, ^21^ especially in the early breast cancer patients with long term survival. Considering the safety and appropriate cosmetic outcome of IOERT from the present study and previous studies by Fastner et al^9^ and Ivaldi et al,^8^ IOERT can be recommended as one of the feasible methods of tumor bed boost dose in patients with breast cancer who are candidate for BCS and adjuvant HWBI. The combination of BCS + IOERT followed by HWBI leads to a considerable shorter treatment time. Decreasing the overall treatment time is considered important especially at this time of Coronavirus disease 2019 (COVID‐19) pandemic since patients with malignancies are at higher risk of mortality if infected by COVID‐19.^22^, ^23^ Using shorter treatment time in addition to deploying appropriate personal protective equipment and adopting appropriate sterilization protocols that lead to decreased exposure to the virus is now recommended by international guidelines in the era of COVID‐19 for all the cancer patients that attend the radiation therapy facilities.^24^, ^25^, ^26^

This trial has a number of limitations. One‐year follow‐up is too short for detecting the in breast recurrence especially in patients with early breast cancer and this could limit drawing inference about clinical efficacy of IOERT from the findings of the present study. However, data derived from previous Phase II/III trials shows low local recurrence rate by using IOERT.

## 5 CONCLUSION

In conclusion, the present study showed that applying high dose boost to the tumor bed using IOERT followed by HWBI regimen has a comparable acute and late treatment toxicity profile in comparison to the conventional whole breast irradiation.

## AUTHOR CONTRIBUTIONS

P. F.: Conceptualization; investigation; supervision; validation; writing‐review & editing. N. N.: Conceptualization; investigation; project administration; supervision; validation; writing‐review & editing. S. R. M.: Conceptualization; data curation; software; supervision; visualization. B. J. and S. A. J. Formal analysis; investigation; methodology; visualization; writing‐original draft.

## CONFLICT OF INTEREST

Authors declare there is no conflict of interest.

## ETHICAL STATEMENT

The study protocol was approved by the Research Ethics Committee of Medical School of Iran University of Medical Sciences (IR.IUMS.FMD.REC.1398.319). Also, the study has been registered on Iranian Registry of Clinical Trials (IRCT20180919041070N2). Undersigned informed consent was obtained from all patients prior to the enrollment.

## Data Availability

All data generated and analyzed during this study can be accessible through direct communication with corresponding author and agreement of all research team members.
